# Pasture diversity and regenerative management shape ruminant gut microbiome dynamics within a real multi-year farming system

**DOI:** 10.3389/fmicb.2026.1928530

**Published:** 2026-08-31

**Authors:** Upulika Jayaneththi, Nicholas W. Sneddon, Lucy L. Burkitt, Paramsothy Jeyakumar, Lisanne M. Fermin, Christopher W. N. Anderson, Daniel J. Donaghy

**Affiliations:** 1 School of Agriculture and Environment, Massey University, Palmerston North, New Zealand; 2 Department of Agricultural Engineering and Soil Science, Faculty of Agriculture, Rajarata University of Sri Lanka, Mihintale, Sri Lanka; 3 School of Veterinary Science, Massey University, Palmerston North, New Zealand

**Keywords:** 16S rRNA sequencing, diverse pastures, pasture management, regenerative agriculture, ruminant gut microbiome, temporal dynamics

## Abstract

The ruminant gut microbiome is central to feed conversion efficiency, nutrient utilisation, and animal health, yet how pasture diversity and management shape its composition over time under real, working farm conditions remains poorly understood, as most previous studies have relied on short-term or more tightly controlled comparisons. A multi-year study (2022–2025) investigated gut microbial dynamics in cattle and sheep grazing on different pasture management systems; standard pastures under contemporary management (Std-Con), standard pastures under regenerative management (Std-Reg), diverse pastures under contemporary management (Div-Con), and diverse pastures under regenerative management (Div-Reg) at Massey University, Palmerston North, New Zealand, using 16S rRNA gene amplicon sequencing of faecal DNA. Microbial relative abundance remained broadly stable overall, with notable seasonal fluctuations. *Faecalibacterium* spp. were the most abundant taxon overall in both hosts, though its relative abundance varied seasonally and dropped below detection in cattle under the Std-Con treatment during winter (August 2023), where *Bacteroides* spp. became dominant instead. Alpha diversity was also stable across treatments for both hosts, however, a sharp decline was observed in Std-Con (1.6 ± 0.27) and Div-Con (1.6 ± 0.21) during winter (August 2023), while Div-Reg remained higher (2.3 ± 0.2) in cattle. Significant temporal signals were also detected in alpha diversity, with a peak across all treatments in cattle in spring (October 2022). In sheep, both regenerative treatments were higher in spring (November 2023) (Std-Reg: 2.59 ± 0.076, Div-Reg: 2.52 ± 0.077). Pairwise beta-diversity comparisons revealed significant divergence in microbial community composition between Std-Con and Div-Reg, most pronounced in cattle from December 2024 to April 2025 (*p* = 0.049–0.015) and in sheep from August to October 2023 (*p* = 0.003–0.001). These findings demonstrate that, under real, multi-year grazing conditions, seasonal effects played a greater role in shaping gut microbial dynamics than pasture treatment alone, with regenerative management buffering a marked winter decline in cattle. Future studies directly linking functional gene expression, forage-quality, and animal production data to microbiome composition within the same sampling framework are needed to determine whether these compositional shifts translate into measurable benefits for ruminant health and productivity.

## 1 Introduction

The gut microbiome is a key determinant of ruminant productivity, directly influencing feed conversion efficiency, nutrient absorption, and host health outcomes (Balasubramanian and Liu, 2024). In grazing livestock, microbial community composition determines the efficiency with which dietary fibre and other plant-derived substrates are fermented to short-chain fatty acids, particularly butyrate, propionate, and acetate, which collectively account for a substantial proportion of the host’s metabolisable energy supply (Barathan et al., 2024). The composition and stability of these communities are shaped by feed type, dietary diversity, environmental conditions (Faniyi et al., 2019), and management practices within grazed pastoral systems (Song et al., 2020). Understanding how pasture management decisions alter gut microbial structure, therefore has direct practical relevance for optimising animal nutrition and livestock production outcomes (Jayaneththi et al., 2026a).

In New Zealand, contemporary pasture systems typically rely on standard pastures dominated by a limited range of species, most commonly a perennial ryegrass-white clover combination (Cunningham et al., 1994; Gilliland et al., 2007). These pastures are intensively managed with short-rotation grazing, and fertiliser and chemical inputs aimed at maximising forage yield and seasonal growth (Gray, 2024). However, limited plant diversity in these systems narrows the nutrients and bioactive compounds available to grazing ruminants (Rochfort et al., 2008), potentially constraining the diversity and functionality of gut microbial communities (Henderson et al., 2015).

Regenerative agriculture encompasses a suite of management practices designed to enhance ecological function, improve soil health, and support long-term animal productivity (Schreefel et al., 2020). A core principle of regenerative management is increasing plant diversity within pastures, including grasses, legumes, and herbs (Merfield, 2019). The inclusion of legumes and herbs enhance nitrogen fixation, improve forage quality, and introduce secondary metabolites such as condensed tannins (Phelan et al., 2015; Ramírez-Restrepo and Barry, 2005). These compounds can modulate rumen fermentation, suppress pathogenic microbes, and promote beneficial microbial taxa in the ruminant gut (Ramírez-Restrepo and Barry, 2005). Diverse swards also provide a broad range of macro and micronutrients, collectively creating a more complex substrate environment in the gut (Rawal et al., 2024). This complexity fosters the development of a stable and diverse gut microbiome capable of efficiently degrading plant biomass, synthesising essential nutrients, and resisting parasitic infection and environmental stress (Rawal et al., 2024). Additionally, regenerative systems often integrate rotational grazing with longer pasture recovery intervals, creating pastures with a broader range of plant species, canopy heights and growth forms (Grelet et al., 2021). Such changes in sward structure and composition may modify feed quality and intake behaviour in ways that influence gut microbial composition, consistent with evidence linking pasture exposure to hindgut microbiome composition in grazing livestock (Jefferson et al., 2025).

Ruminants grazing on diverse pastures under rotational grazing have exhibited greater microbial diversity and improved production outcomes, including greater yields of milksolids, milk protein, and milk fat, along with enhanced live weight gain and body condition score, compared with animals grazing on monocultures or simple ryegrass–white clover swards (Carmona-Flores et al., 2020; Kenyon et al., 2010; Totty et al., 2013). In these trials, diverse pastures contained at least five to six sown species, typically including forage herbs such as chicory or plantain together with one or more clover species, whereas low-diversity pastures were grass monocultures or simple ryegrass-white clover mixtures. Although these studies do not explicitly describe their systems as regenerative, the combination of diverse swards and rotational grazing aligns with key elements of regenerative grazing practice. For example, in the USA, Carmona-Flores et al. (2020) reported that dairy cows rotationally grazing six-species pastures produced significantly greater daily yields of milksolids and milk protein than cows grazing ryegrass-white clover pastures. In New Zealand, Totty et al. (2013) found that cows offered five-species diverse pastures under rotational grazing had significantly higher milk yield (approximately 10% on average across the grazing season) than cows on standard ryegrass-white clover swards.

Microbial diversity resulting from diets of species-rich pastures has been linked to improved feed conversion efficiency and reduced enteric methane emissions from ruminants (Ramírez-Restrepo and Barry, 2005; Waghorn et al., 2002). Waghorn et al. (2002) demonstrated that sheep fed a condensed-tannin-containing forage produced 16% less methane than those fed ryegrass-white clover pasture, illustrating the potential of condensed-tannin-rich forages, such as those found in diverse pastures, to reduce methane emissions.

Despite growing interest in diverse pastures and regenerative grazing systems, multi-year, real farm-scale studies assessing how pasture diversity and management practices jointly influence ruminant gut microbiomes across host species and seasons remain limited. Most previous research has focused on short-term or single-system comparisons, providing only partial insight into microbiome stability and temporal dynamics under practical grazing conditions.

Recent advances in high-throughput multi-omics techniques now enable more detailed investigation of microbial composition, abundance, and diversity dynamics in the ruminant gut (Deusch et al., 2015).

Hindgut (colonic) microbial communities, typically characterised using faecal samples, provide a practical assessment of gut microbial ecology because they integrate signals from the upper gut (Hao et al., 2025). These communities differ from, but are related to, the rumen microbial community.

In the present study, 16S rRNA gene amplicon sequencing was employed to characterise and compare the hindgut bacterial communities, reflecting the broader gut microbiome of dairy cattle and sheep grazing on diverse and standard pastures under regenerative and contemporary management, within a working commercial-scale farm system over three to 4 years. Functional roles of key taxa were inferred from published literature and ecological associations. It was hypothesised that diverse pastures, particularly under regenerative management, would support greater bacterial abundance, diversity, and distinct community composition compared with standard pastures under contemporary management in both cattle and sheep.

## 2 Materials and methods

### 2.1 Experimental design and site description

Field experiments were conducted at Massey University’s Dairy Farm 1 (S 40° 22′50″, E 175° 36′55″) and Pasture and Crop Research Unit (PCRU) (S 40° 23′20″, E 175° 37′0″), Palmerston North, New Zealand. These sites are part of the Whenua Haumanu project, a comprehensive seven-year programme initiated in 2022 that compares diverse and standard pastures under regenerative and contemporary management in dairy and sheep systems (Jayaneththi et al., 2025; Jayaneththi et al., 2026b). Animal ethics approval was granted by the Massey University Animal Ethics Committee under protocols of AEC22/17, AEC22/18, AEC24/31, and AEC24/47.

At the dairy site, three cattle herds, with representation from three common breeds (Holstein-Friesian, Jersey, and Holstein-Friesian Jersey crossbred), were rotationally grazed on a 12-ha farmlet. Cows were mature adults (2–9 years old) with comparable age distributions across treatments, with each herd allocated to one of three treatments: standard pastures under contemporary management (Std-Con), diverse pastures under regenerative management (Div-Reg), and diverse pastures under contemporary management (Div-Con). A standard pasture under regenerative management (Std-Reg) treatment was not included in the cattle experiment, reflecting the practical constraints of implementing treatments within a working commercial farm system. The Std-Con treatment comprised diploid and tetraploid perennial ryegrass (*Lolium perenne* L.), large- and medium/large-leaved white clover (*Trifolium repens* L.) and red clover (*Trifolium pratense* L.), plantain (*Plantago lanceolata* L.) managed according to DairyNZ guidelines (DairyNZ, 2025) with contemporary use of mineral fertilisers and agrichemicals as needed.

The diverse pasture treatments (Div-Reg and Div-Con) were established with 18 sown species. However, only diploid and tetraploid perennial ryegrass, meadow fescue (*Festuca pratensis* Huds.), cocksfoot (*Dactylis glomerata* L.), red clover, large- and medium/large-leaved white clover, chicory (*Cichorium intybus* L.), and plantain persisted across both treatments at the final pasture assessment in April 2025 (Jayaneththi et al., 2026b).

The Div-Reg treatment was managed with minimal fertiliser, amendments including fish hydrolysate, seaweed, humates, fulvic acid and lime flour, no agrichemicals, increased mechanical weed control, longer grazing intervals, and higher post-grazing residuals. In contrast, the Div-Con treatment was managed according to DairyNZ guidelines (DairyNZ, 2025), with contemporary use of mineral fertilisers and agrichemicals.

At PCRU, up to 40 Romney ewes (3–5 years old) were rotationally grazed on 3-ha farmlets across four treatments: standard pastures under contemporary management (Std-Con), standard pastures under regenerative management (Std-Reg), diverse pastures under contemporary management (Div-Con), and diverse pastures under regenerative management (Div-Reg). Diverse pastures were initially established with 19 sown species, but only diploid and tetraploid perennial ryegrass, meadow fescue, cocksfoot, red clover, and small- and medium/large-leaved white clover, chicory, and plantain persisted at the final assessment in November 2024 (Jayaneththi et al., 2026b). Contemporary management followed Beef + Lamb NZ guidelines (Beef + Lamb New Zealand, 2018), and regenerative practices mirrored those implemented at the dairy farmlets.

Pasture species compositions and Shannon diversity details for both cattle and sheep systems are reported in Jayaneththi et al. (2026b).

### 2.2 Stocking rates and grazing management

Stocking rates varied annually and by treatment (Table 1) due to evolving pasture establishment and growth patterns. Rotational lengths also differed by management approach, with regenerative treatments employing longer rest periods across all seasons (Table 2).

**Table 1:** Stocking rates for dairy and sheep farmlet treatments across study years (2022–2025).

Farmlet system	Treatment	2022–2023	2023–2024	2024–2025
Dairy (cows/ha)	Std-Con	2.0	2.5	2.5
	Div-Con	2.0	2.5	2.0
	Div-Reg	2.0	2.5	2.0
Sheep (ewes/ha)	Std-Con	15.0	14.0	14.0
	Std-Reg	15.0	12.0	14.0
	Div-Con	15.0	13.0	14.0
	Div-Reg	15.0	12.0	14.0

Std-Con: standard pastures under contemporary management; Std-Reg: standard pastures under regenerative management; Div-Con: diverse pastures under contemporary management; Div-Reg: diverse pastures under regenerative management.

**Table 2:** Seasonal rotational lengths under contemporary and regenerative management in dairy and sheep farmlets.

Farmlet system	Season	Rotation length (days)	
		Contemporary	Regenerative
Dairy	Spring	20–30	40–50
	Summer	30–40	50–60
	Autumn	40–60	60–80
	Winter	40–60	60–80
Sheep	Spring	12–24	24–48
	Summer	30–40	50–60
	Autumn	50–70	80–100
	Winter	60–75	80–100

### 2.3 Sample collection

Faecal samples were collected from 10 pre-nominated grazing dairy cows per treatment, with occasional substitutions due to illness or removal from the study, and 10 randomly selected ewes per treatment via rectal palpation (Ransom et al., 2002). Pre-nominated cows were selected to balance groups for age and breed. Cow sampling was conducted from August 2022 to April 2025, while ewe sampling commenced from March 2023 to November 2024. All faecal samples were immediately preserved in RNAlater (Thermo Fisher Scientific, Lithuania) to stabilise nucleic acids and prevent degradation (Camacho-Sanchez et al., 2013). Samples were stored at −20 °C until further analysis.

### 2.4 DNA extraction and 16S rRNA gene sequencing

Total DNA was extracted from 0.2 g of each faeces sample using the Presto™ Stool DNA Extraction Kit (Geneaid, Taiwan) following the manufacturer’s instructions. DNA purity was assessed with a NanoDrop 2000 spectrophotometer (Thermo Fisher Scientific, USA). NanoDrop A260/A280 ratios ranged from 1.85 to 2.03, while A260/A230 ratios ranged from 1.80 to 2.05.

Amplicon libraries covering the V3-V4 region of the bacterial 16S rRNA gene were generated using a single-step PCR protocol with primers 341F and 805R. Sequencing was subsequently carried out on an Illumina MiSeq platform (Illumina, USA), applying the 500-cycle V2 kit to produce 2 × 250 bp paired-end reads.

### 2.5 Bioinformatic analysis

Anaconda3 (v25.3.0) environment was used to run Python (v3.12.3) for bioinformatic analyses.

#### 2.5.1 Sequence quality control and taxonomic classification

Raw 16S sequence data were quality-assessed with FastQC (Andrews, 2010) and trimmed at a Phred score of 20 using SolexaQA++ (Cox et al., 2010). Taxonomic classification was performed using Kraken2 (v2.1.3) (Wood et al., 2019), with abundance re-estimation using Bracken (v2.9) (Lu et al., 2017), against the Greengenes 16S reference database (release 13_5). Although Greengenes 13_5 (May 2013) predates more recent databases and its limitations are acknowledged, it continues to be used in current microbiome research (Cornejo-Granados et al., 2025; Chornchoem et al., 2025; Licata et al., 2025). To validate the taxonomic assignments, sequence reads were re-classified against the SILVA database as a spot-check, and the same dominant genus-level taxa were identified using both reference databases. Taxonomic classification profiles for representative samples from selected treatments were visualised using the Pavian Shiny application.^1^

Taxonomic assignment to species-level resolution was included in this descriptive and comparative study because species-level profiles can provide additional ecological interpretation of temporal dynamics and treatment effects beyond genus-level summaries (Escapa et al., 2020; Graham et al., 2025; Gwak and Rho, 2020; Wang et al., 2025). However, we acknowledge that short-read 16S V3-V4 sequence data have limited power to distinguish among closely related species (Almeida et al., 2018). Therefore, species-level assignments derived in this study are considered indicative rather than definitive (Gwak and Rho, 2020), and the primary interpretation of microbial community dynamics was based on genus-level classifications.

Sequencing depth varied across samples, ranging from 0.02 to 0.21 million reads per sample for 16S rRNA gene sequencing.

#### 2.5.2 Taxa relative abundance and dominance thresholds

Taxonomic classifications were filtered to retain taxa with a Bracken-estimated relative abundance (fraction_total_reads) of at least 5% within each sample replicate as candidate taxa for dominance classification (McHugh et al., 2025). For each replicate, Bracken-estimated read counts (new_est_reads) for all candidate taxa were summed, and individual taxon counts were converted to replicate-level relative abundances. Treatment-level mean relative abundances were then calculated by averaging replicate values for each taxon. Taxa with a treatment-level mean relative abundance exceeding 50% of the highest mean abundance observed within that treatment were classified as dominant taxa (Gray et al., 2021). Taxa that did not meet either the 5% Bracken threshold or the 50% treatment-level dominance threshold were classified as low-abundance taxa. These relative thresholds were applied to retain taxa that are consistently abundant within treatments while avoiding over-interpretation of very low-abundance species (Neu et al., 2021; Weiss et al., 2017).

#### 2.5.3 Microbial diversity metrics

Alpha and beta diversity were calculated from the original, unfiltered Bracken output files using KrakenTools (v1.2), independent of the dominant-taxa filtering described in Section 2.5.2. Alpha diversity was assessed using the Shannon index, which reflects both taxonomic richness and evenness within a sample (Roswell et al., 2021). Beta diversity, calculated as Bray–Curtis dissimilarity, was used to compare overall microbial community composition across treatments and time points (Kers and Saccenti, 2022).

### 2.6 Statistical analysis

Statistical analyses were performed in R (v4.3.3). Differences in dominant taxon relative abundances and alpha diversity among treatments were assessed using one-way ANOVA, followed by Tukey’s post-hoc comparisons using the multcompView (v0.1.10) package. Breed and age effects were also assessed jointly in cattle using two-way ANOVA, with neither found to be significant. These effects were not tested in sheep, as ewes were of a comparable age range with uniform flock management.

For sheep, where the experimental design included all four treatment combinations (Std-Con, Std-Reg, Div-Con, Div-Reg), a two-way ANOVA was additionally conducted to evaluate the effects of pasture diversity, management, and their interaction on Shannon diversity using the car (v3.1.5) package. As each farmlet was managed as an integrated system, with stocking rate, fertiliser input, and pasture management varying across treatments and years, this analysis should be interpreted as an exploratory comparison among four management systems rather than a controlled test of independent factors. This equivalent analysis was not possible for cattle because the experimental design did not include a Std-Reg treatment.

Associations among species relative abundances within individual treatments were evaluated using Spearman’s rank correlation coefficient (r), with *p*-values adjusted for multiple comparisons using the false discovery rate (FDR) correction. Correlation strength was interpreted based on the absolute value of Spearman’s *r* (Akoglu, 2018). Only correlations within the strongest 25% of observed associations were included in subsequent network analyses. Co-occurrence networks were constructed using the igraph (v2.1.4) package, with clusters of strongly correlated species identified using the Louvain community detection algorithm and visualised using ggraph (v2.1.1).

Heatmaps of taxon relative abundance were generated using tidyverse (v2.0.0) and pheatmap (v1.0.12). Dominant taxa consistently detected across sampling time points were selected for temporal analysis. Temporal variation in the relative abundance of these taxa and alpha diversity metrics was analysed using linear mixed-effects models (LMMs), with estimated marginal means (EMMs) and Tukey-adjusted pairwise comparisons implemented using the lme4 (v1.1.36), lmerTest (v3.1.3), emmeans (v1.11.2), and multcomp (v1.4.28) packages. Temporal trends were visualised using ggplot2 (v3.5.1), with locally estimated scatterplot smoothing (LOESS) curves overlaid to illustrate longitudinal patterns.

Differences in beta diversity among treatments and sampling time points were assessed using permutational multivariate analysis of variance (PERMANOVA) with 999 permutations, implemented using the vegan (v2.6.10) and pairwiseAdonis (v0.4.1) packages. Changes in R^2^ values, representing the proportion of community variation explained by treatment, were calculated between treatment pairs over time to support interpretation of temporal shifts in microbial community structure.

## 3 Results

### 3.1 Gut microbiome composition and relative abundance

**Figure 1:**
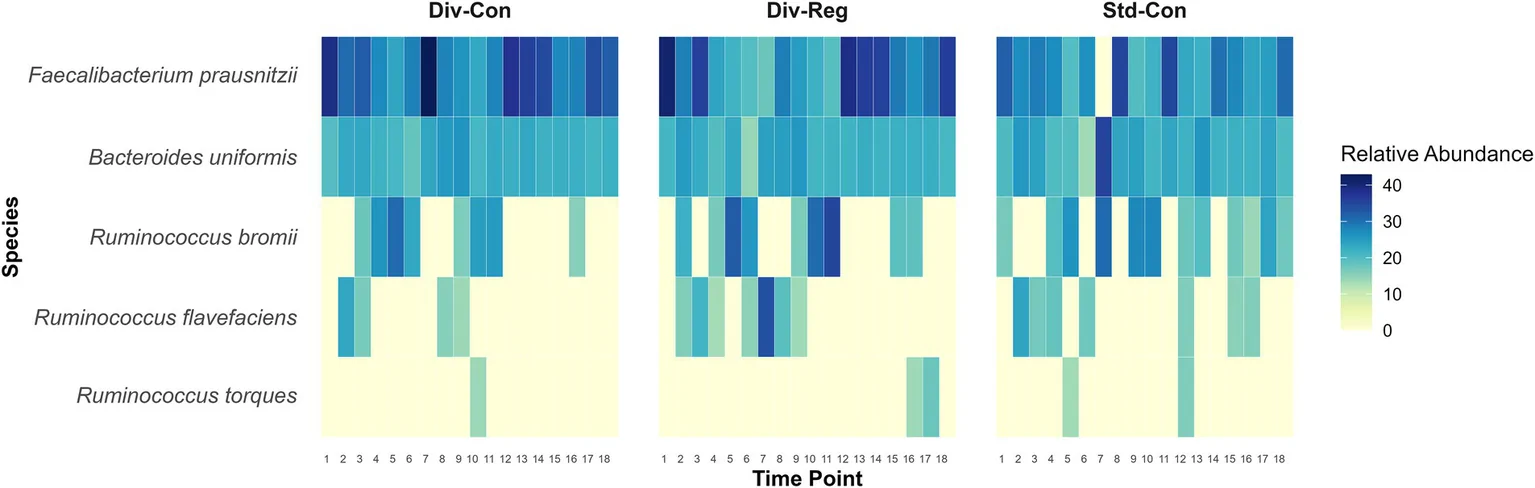
Temporal dynamics of the cattle gut bacterial microbiome across experimental treatments. The heatmap illustrates the relative abundance (0%–40%) of the five predominant bacterial taxa under three treatments: diverse pastures under contemporary management (Div-Con); diverse pastures under regenerative management (Div-Reg); standard pastures under contemporary management (Std-Con). Relative abundance was measured at 18 time points from August 2022 to April 2025. Time points are numbered (1–18) and correspond to specific months [1: August 2022; 2: October 2022; 3: November 2022; 4: December 2022; 5: February 2023; 6: April 2023; 7: August 2023; 8: October 2023; 9: November 2023; 10: December 2023; 11: February 2024; 12: April 2024; 13: August 2024; 14: October 2024; 15: November 2024; 16: December 2024; 17: February 2025; 18: April 2025]. Row labels indicate bacterial species, and columns represent sampling time points. Colour intensity reflects abundance: Dark blue (high, 35≥40%), intermediate blue (moderate, 25%–35%), light yellowish green (low, 5%–15%), and yellow (minimal, <5%). Species-level assignments are indicative rather than definitive, given the resolution limits of short-read 16S data.

**Figure 2:**
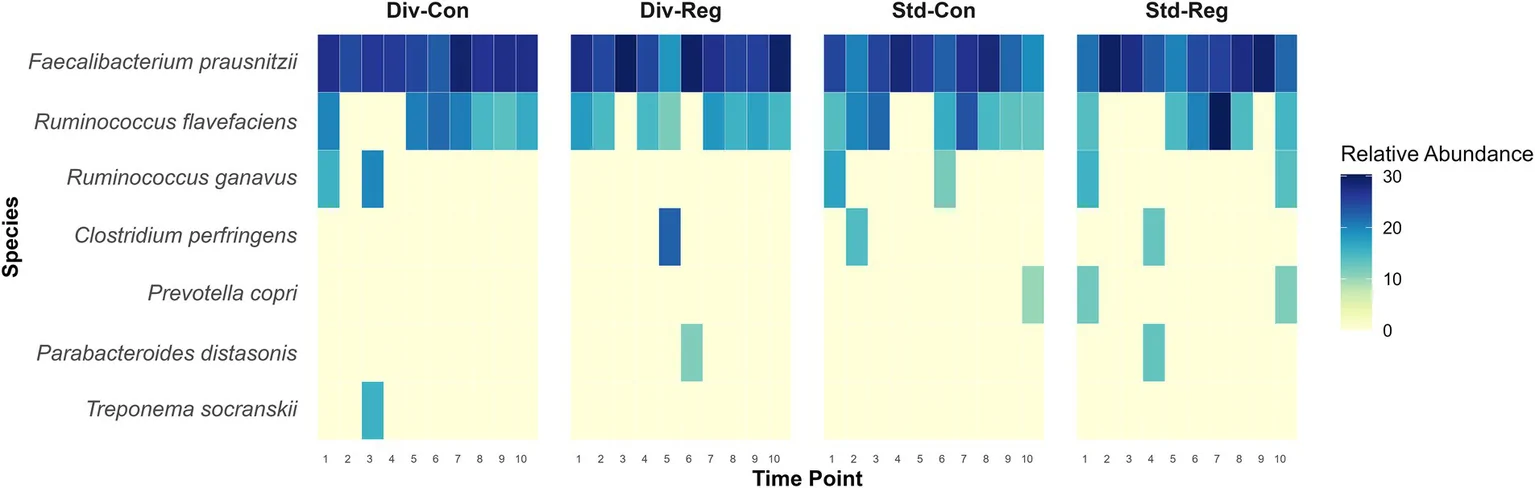
Temporal dynamics of the sheep gut bacterial microbiome across experimental treatments. The heatmap illustrates the relative abundance (0%–30%) of the seven predominant bacterial taxa under four treatments: Diverse pastures under contemporary management (Div-Con), diverse pastures under regenerative management (Div-Reg), standard pastures under contemporary management (Std-Con), and standard pastures under regenerative management (Std-Reg). Relative abundance was measured at 10 time points from March 2023 to November 2024 [1: March 2023; 2: June 2023; 3: August 2023; 4: October 2023; 5: November 2023; 6: March 2024; 7: June 2024; 8: August 2024; 9: October 2024; 10: November 2024]. Colour intensity indicates relative abundance: Dark blue (high, 25≥30%), intermediate blue (moderate, 15%–25%), light blue/green (low, 5%–15%), and yellow (minimal, <5%). Species-level assignments are indicative rather than definitive, given the resolution limits of short-read 16S data.

Heatmap analyses revealed clear temporal and treatment-dependent shifts in the gut microbiome of both cattle and sheep (Figures 1, 2). Among the examined variables, treatment and sampling time exerted the strongest influence on microbiome composition and relative abundance, while breed and age showed no significant effect in cattle. Across all treatments, *Faecalibacterium* spp., indicated as *Faecalibacterium prausnitzii* consistently emerged as the dominant species in both ruminants (Figures 1, 2).

#### 3.1.1 Cattle

**Figure 3:**
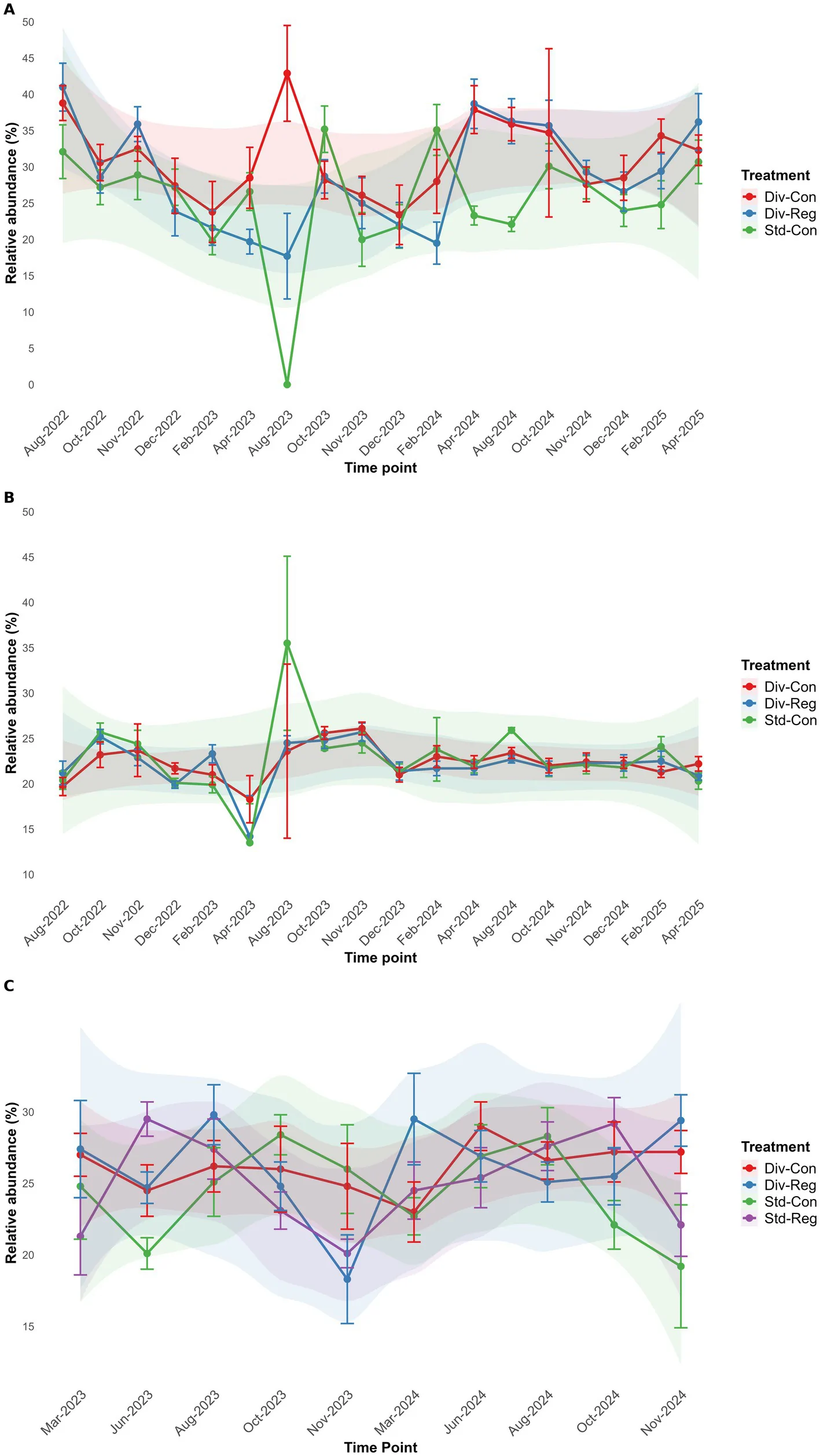
Relative abundance of predominant bacterial species in the ruminant gut microbiome across treatments. **(A)** *Faecalibacterium prausnitzii* and **(B)** *Bacteroides uniformis* in the cattle gut across 18 sampling time points from 2022 to 2025. **(C)** *Faecalibacterium prausnitzii* in the sheep gut across 10 sampling time points from 2023 to 2024. Species-level assignments are indicative rather than definitive, given the resolution limits of short-read 16S data.

The relative abundance of *F. prausnitzii* remained largely consistent across treatments over the three-year period, except in August 2023 when it was significantly (*p* < 0.05) higher in Div-Con (42.9 ± 6.6%) compared to Div-Reg (17.7 ± 5.9%), and undetectable in Std-Con (Figures 1, 3A). Temporal analysis within treatments revealed significant peaks (*p* < 0.05) in October 2023, February 2024, and April 2025 with Std-Con; Div-Reg peaked in August 2022, and Div-Con in August 2023 (Supplementary Table S1).

*Bacteroides* spp., indicated as *Bacteroides uniformis* maintained a generally consistent abundance across treatments, with only small changes over time (Figures 1, 3B). Std-Con peaked in August 2023 (*p* < 0.05), Div-Reg remained consistently high across most time points, and Div-Con peaked in November 2023 (*p* < 0.05) (Supplementary Table S1). Other *Ruminococcus* spp. appeared intermittently across treatments, with notable peaks in Div-Reg (Figure 1).

#### 3.1.2 Sheep

*Faecalibacterium prausnitzii* remained the predominant species, with no significant differences detected among treatments (Figures 2, 3C). Temporal analysis revealed moderate fluctuations in Std-Con, *F. prausnitzii* reached its lowest (*p* < 0.05) abundance in November 2024 and peaked in October 2023. The Div-Reg treatment maintained consistently high values, with significantly higher abundances (*p* < 0.05) in August 2023, March 2024, and November 2024, and a significant dip in November 2023 (*p* < 0.05) (Supplementary Table S2). Relative abundance values of Div-Con and Std-Reg were relatively stable during the study period (Supplementary Table S2). Other taxa such as *Ruminococcus* spp., *Prevotella* spp., *Parabacteroides* spp., and *Treponema* spp., were intermittently detected but showed no consistent trends across treatments (Figure 2).

### 3.2 Microbial correlation networks and taxonomic profiling

**Figure 4:**
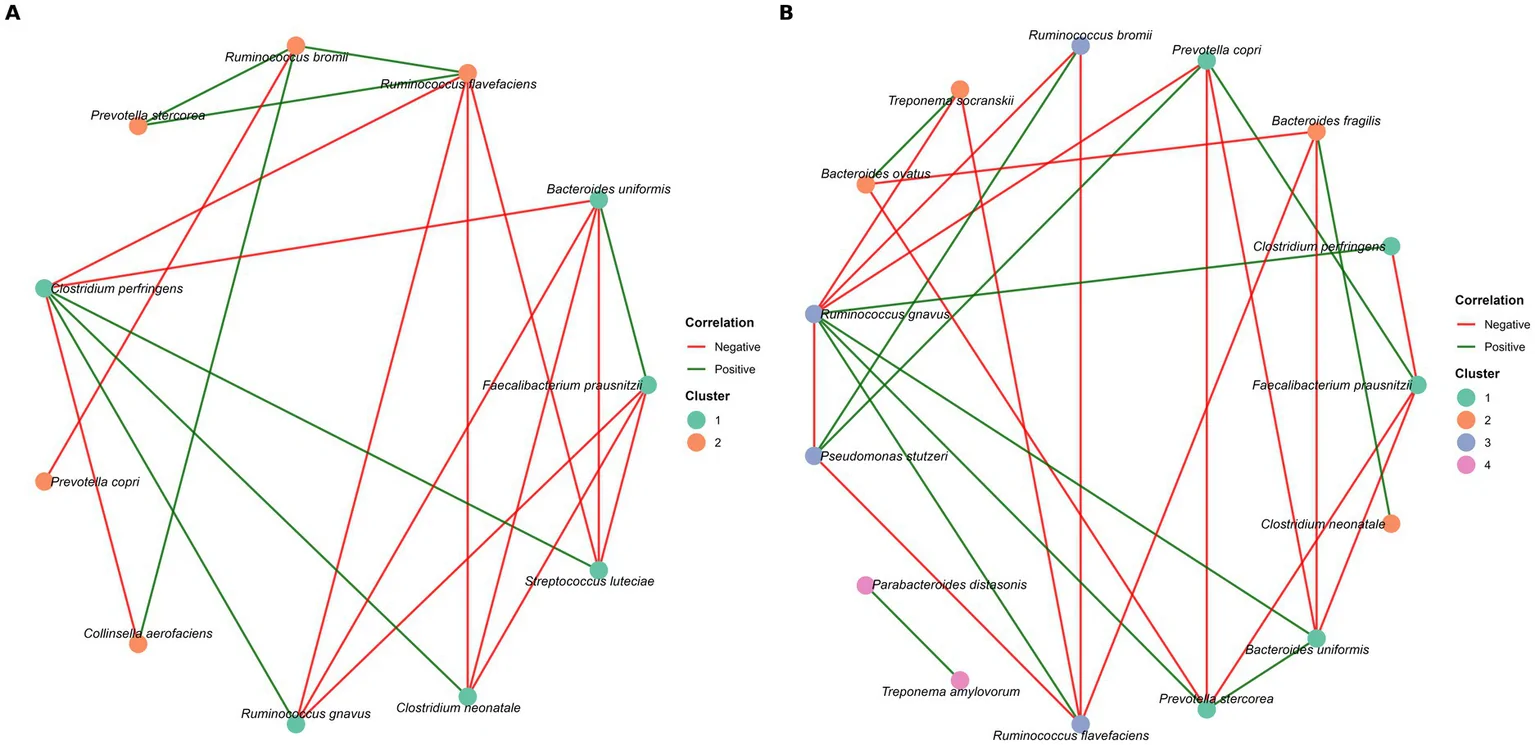
Spearman correlation network of microbial taxa in August 2023. **(A)** Cattle gut microbiome in diverse pastures under contemporary management (Div-Con) and **(B)** sheep gut microbiome in diverse pastures under regenerative management (Div-Reg). Nodes show different bacterial taxa, coloured by clusters found using the Louvain method. Green edges represent positive correlations, and red edges represent negative ones; only the top 25% strongest correlations are shown. Species-level assignments are indicative rather than definitive, given the resolution limits of short-read 16S data.

Exploratory microbial correlation networks were constructed to identify potential co-occurrence patterns among dominant gut taxa. No correlations reached statistical significance after FDR correction. Accordingly, all network results are presented as exploratory and should not be interpreted as evidence of definitive microbial interactions. Networks were constructed for treatments and sampling time points characterised by the highest relative abundance of *F. prausnitzii*, specifically Div-Con in cattle and Div-Reg in sheep during August 2023 (Figures 3A,C). Network analysis revealed clusters of co-occurring taxa, highlighting patterns of association among dominant bacterial species within each treatment (Figures 4A,B).

In cattle, a moderate positive correlation was observed between *F. prausnitzii* and *B. uniformis* (*r* = 0.6, FDR = 0.9, *p* = 0.2), while *B. uniformis* and *C. perfringens* exhibited a strong negative association (*r* = −0.9, FDR = 0.3, *p* = 0.1). In sheep, a moderate positive correlation was detected between *F. prausnitzii* and *P. copri* (*r* = 0.6, FDR = 0.5, *p* = 0.2), and a strong negative correlation between *F. prausnitzii* and *C. perfringens* (*r* = −0.7, FDR = 0.5, *p* = 0.3). These represented the highest correlation coefficients observed in the dataset. Because the analysis was based on a limited number of samples from a single treatment per species, these patterns should be regarded as exploratory and do not allow firm conclusions about pasture management effects.

**Figure 5:**
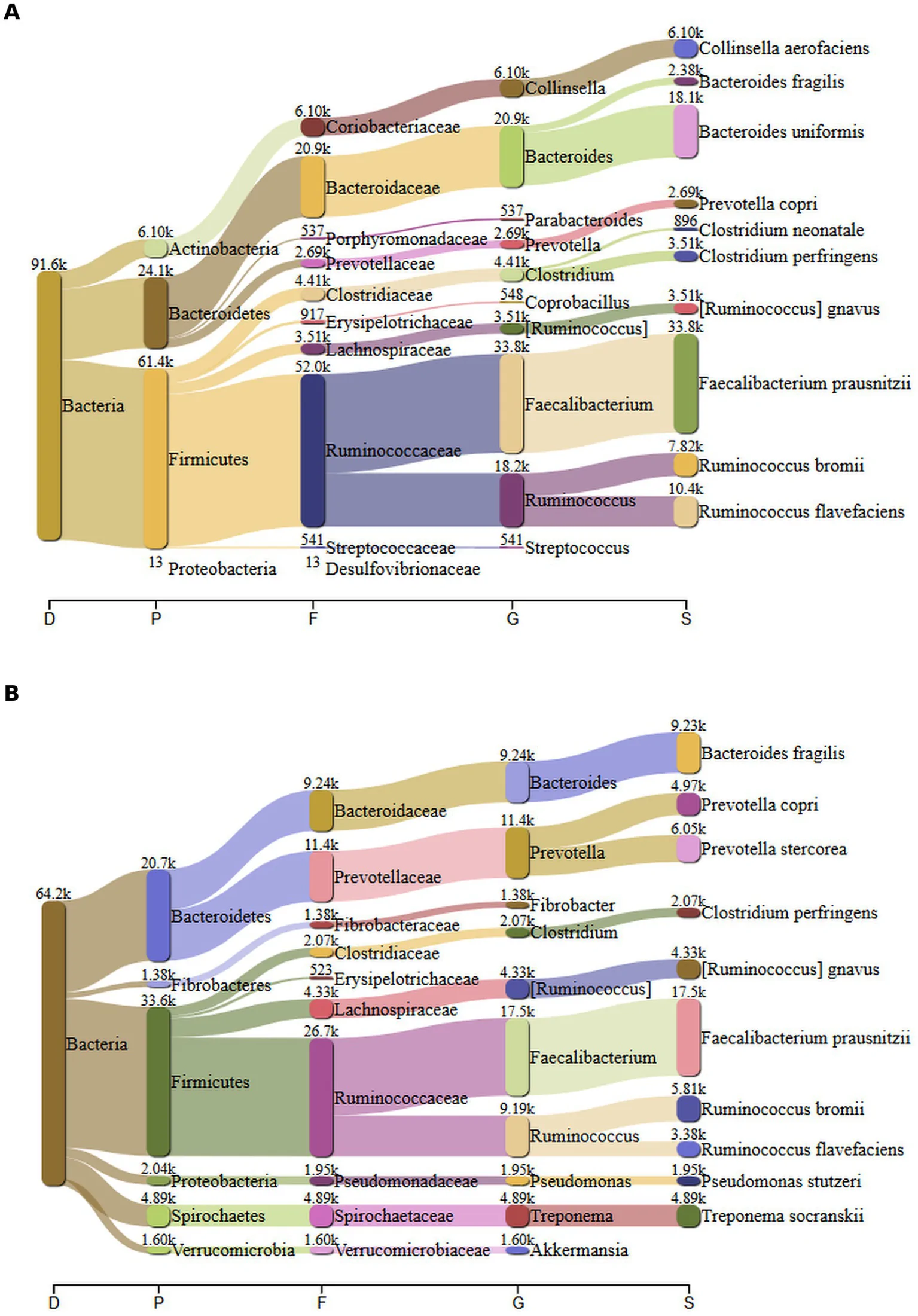
Pavian visualisation of taxonomic hierarchies of bacterial communities in representative gut samples from the treatment and time point with the highest relative abundance of *Faecalibacterium prausnitzii* in each host species (August 2023). **(A)** Cattle gut microbiome in Div-Con and **(B)** sheep gut microbiome in Div-Reg in August 2023. Each diagram shows the flow of classified reads through taxonomic levels from domain (left) to phylum, family, genus, and species (right). Connection width reflects relative abundance, with numeric labels indicating thousands (k) of Kraken-classified sequencing reads. Diagrams represent a single representative sample per treatment and reflect individual sample-level taxonomic profiles; taxa shown may therefore differ from the treatment-averaged heatmaps (Figures 1, 2). Species-level assignments are indicative rather than definitive, given the resolution limits of short-read 16S data.

The taxonomic profiles of bacterial communities in representative samples from cattle (Div-Con) and sheep (Div-Reg) hindgut microbiomes in August 2023 were visualised using Pavian Sankey diagrams (Figures 5A,B). Classified sequencing reads were traced through successive taxonomic levels, from phylum to family, genus, and species, with connection widths proportional to the relative abundance of each taxon. These visualisations corroborate abundance analyses, demonstrating that *F. prausnitzii* was the most prevalent species in both ruminants.

### 3.3 Microbiome diversities

#### 3.3.1 Alpha diversity: Shannon index

**Figure 6:**
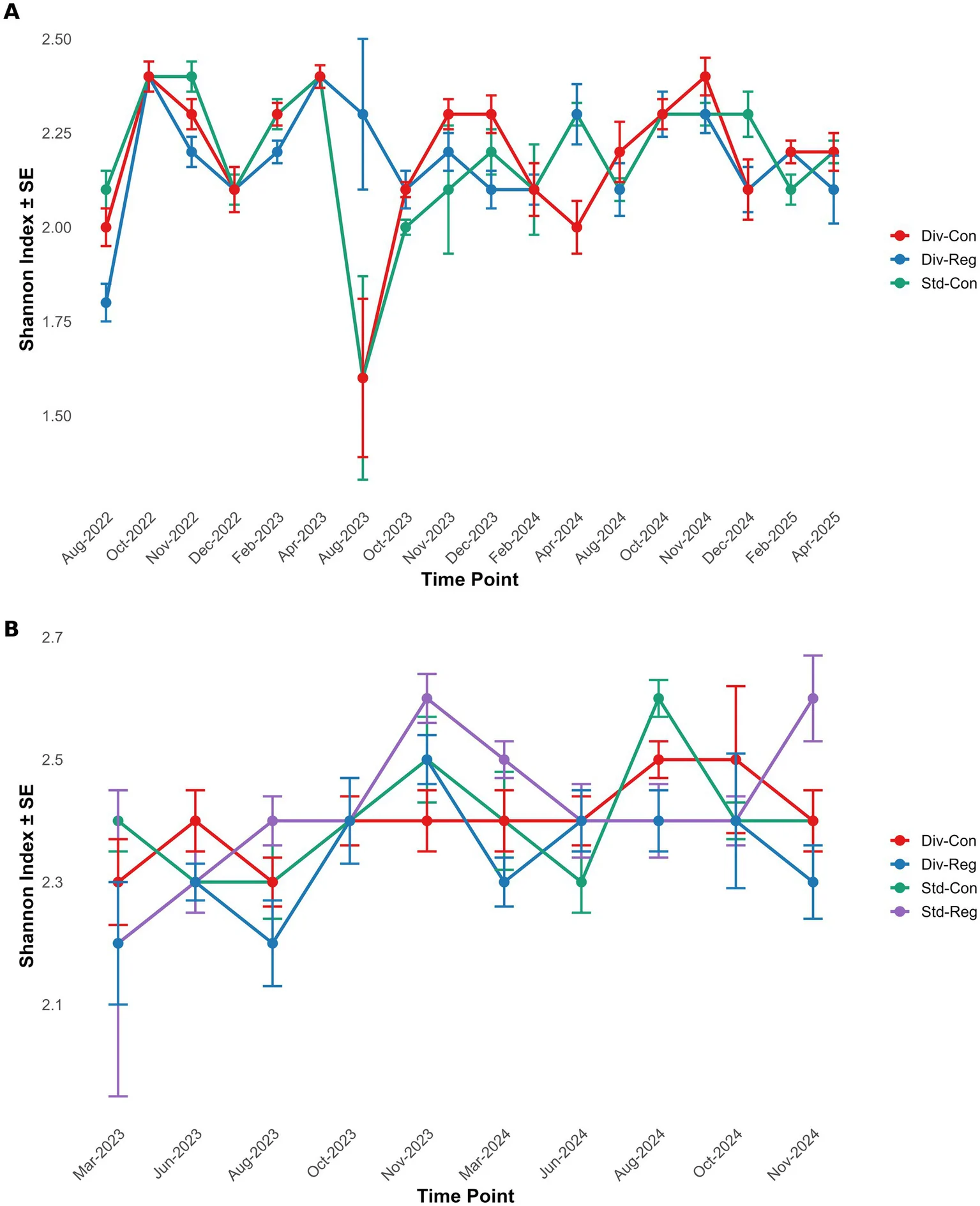
Variation in Shannon index across treatments. **(A)** Cattle gut microbiomes measured at 18 sampling points from 2022 to 2025. **(B)** Sheep gut microbiomes measured at 10 sampling points from 2023 to 2024.

The Shannon index of alpha diversity remained relatively stable across treatments in both cattle and sheep, with no statistically significant differences (Figures 6A,B). However, in cattle, a notable dip in the Shannon index was recorded in Std-Con (1.6 ± 0.27) and Div-Con (1.6 ± 0.21) compared to Div-Reg (2.3 ± 0.2) in August 2023.

Temporal analysis revealed distinct treatment peaks, with significantly higher (*p* < 0.05) diversity in cattle detected in October 2022 across all treatments (Supplementary Table S3). For sheep, no significant temporal differences in alpha diversity were observed within contemporary treatments (Std-Con and Div-Con). In contrast, regenerative treatments (Std-Reg and Div-Reg) showed significantly higher (*p* < 0.05) Shannon diversity in November 2023 (Std-Reg: 2.59 ± 0.076; Div-Reg: 2.52 ± 0.077) (Supplementary Table S4).

An exploratory two-way ANOVA assessing the effects of pasture diversity and management on sheep Shannon diversity revealed no significant main effects of diversity (*p* = 0.231) or management (*p* = 0.519), and no significant diversity × management interaction (*p* = 0.201). The equivalent interaction analysis could not be conducted for cattle due to the incomplete factorial design.

#### 3.3.2 Beta diversity: Bray–Curtis dissimilarity

**Figure 7:**
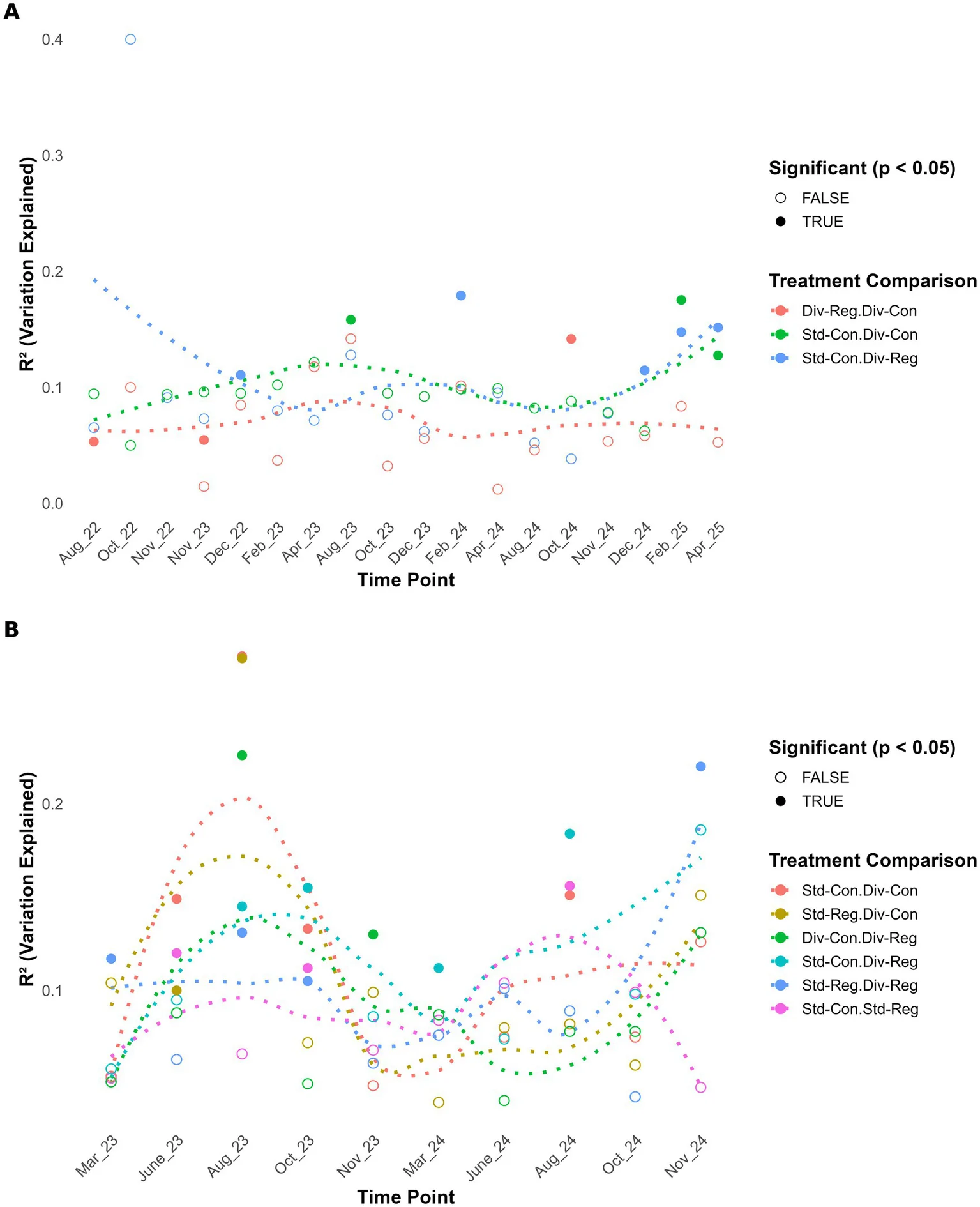
Temporal changes in beta diversity (PERMANOVA, *R*^2^) for pairwise treatment comparisons. **(A)** Cattle gut microbiome measured at 18 sampling points from 2022 to 2025; **(B)** sheep gut microbiome measured at 10 sampling points from 2023 to 2024. LOESS-smoothed lines display the change in *R*^2^ over time for each treatment pair. Solid points identify when treatment effects are statistically significant (*p* < 0.05).

Pairwise comparisons of beta diversity in cattle and sheep gut microbiomes provide a clear overview of the temporal effects exerted by all pasture management treatment combinations on gut microbial community composition (Figures 7A,B). In cattle, more pronounced and significant (*p* < 0.05) differences in beta diversity were detected between Std-Con and Div-Reg, from December 2024 (*p* = 0.049) to April 2025 (*p* = 0.015) (Figure 7A). In sheep, significant treatment effects between Std-Con and Div-Reg were more distinct from August (*p* = 0.003) to October 2023 (*p* = 0.001) (Figure 7B). Most other pairwise comparisons between Std-Con and Div-Reg did not show significant divergence in beta diversity, indicating similar effect on hindgut microbial community composition for most other sampling time points.

## 4 Discussion

### 4.1 Temporal dynamics of dominant taxa

Conducted over 3 years on a working commercial farm, this analysis captures ruminant gut microbiome dynamics under genuine seasonal and inter-annual variation rarely accessible in shorter-term or more tightly controlled trials. Overall microbial abundance in both cattle and sheep hindgut communities remained relatively stable across this period, consistent with longitudinal studies of the ruminant gastrointestinal microbiome more broadly (Huws et al., 2018; Lima et al., 2024), while treatment-related differences were more apparent in the composition and temporal dynamics of dominant microbial taxa.

Diverse pastures, whether managed under contemporary or regenerative systems, generally supported greater relative abundances of key hindgut bacterial taxa in cattle and sheep (Figures 1, 2). Increased dietary plant diversity is widely associated with a more complex and enriched microbiome due to a broader range of fermentable substrates available (Li et al., 2023). Among the dominant taxa, *Faecalibacterium* spp. were indicated as the most abundant fibre-degrading taxon in both cattle and sheep (Figures 3A,C). Members of *Faecalibacterium* spp. ferment dietary fibre to butyrate, a short-chain fatty acid with notable anti-inflammatory properties central to gut health (Wrzosek et al., 2013), consistent with prior isolation and characterisation of *F. prausnitzii* from New Zealand dairy calves (Lim, 2022) and from calves and piglets more broadly (Foditsch et al., 2014).

Distinct seasonal peaks in *Faecalibacterium* spp. were observed in late winter in August 2023 for both ruminants, consistent with broader evidence that seasonal pasture variation drives shift in microbial community composition and volatile fatty acid output in the rumen (He et al., 2024). In sheep, its abundance was consistently stable across all treatments (Figure 3C). In cattle, grazing on Div-Con pastures markedly promoted its proliferation, consistent with the broader principle that increased pasture diversity enhances fermentable fibre availability and supports the growth of key butyrate-producing taxa during fibre degradation (Jerrentrup et al., 2020; Jezequel et al., 2024) and with the greater botanical diversity documented for diverse pasture treatments within this experimental system (Jayaneththi et al., 2026b) (Figure 3A). As direct forage chemical composition data were not collected in this trial, this substrate-availability explanation remains hypothesised.

Regenerative practices, including reduced fertiliser and agrichemical inputs, have been associated with shifts in sward botanical composition, such as reduced legume content, under regenerative relative to contemporary management more broadly (Cosgrove et al., 2024). Within this study, Div-Con and Div-Reg were sown with the same plant species but differed in management input intensity as Div-Con received contemporary mineral fertiliser and agrichemical inputs, whereas Div-Reg was managed with minimal fertiliser and no agrichemicals. This difference in input intensity, rather than species composition, may drive higher fermentable substrate availability, and correspondingly higher *Faecalibacterium* spp. abundance, observed in Div-Con relative to Div-Reg during winter (August 2023). Diverse pastures under higher-input contemporary management may therefore have supported greater pasture biomass and nutrient availability during the winter constraint period, potentially contributing to the persistence of *Faecalibacterium* spp. in Div-Con at this time (Li et al., 2023; Rochfort et al., 2008). However, *Faecalibacterium* spp. were undetectable in Std-Con pastures at this same time point, which may reflect seasonal and nutritional factors overriding the expected effects of contemporary management on standard, lower-diversity pastures specifically. Standard ryegrass-white clover monocultures are particularly susceptible to winter declines in pasture quality, where reduced biomass production and changes in nutrient availability may limit the supply of substrates supporting this genus (Nielsen et al., 2003). These patterns are consistent with seasonal dietary shifts and reduced forage availability reported to influence ruminant gut microbial communities during cold-season transitions (Cui et al., 2023). This decline coincided with an unusually cold period with mean temperature in August 2023 was 8.0 °C (minimum 2.8 °C), approximately 3 °C lower than the same month in the preceding and following years (August 2022: 11.0 °C; August 2024: 10.6 °C; NIWA, 2025). The unusually cold conditions may therefore have contributed to altered pasture growth and seasonal dietary constraints, potentially intensifying the observed decline in *Faecalibacterium* spp. proliferation.

Other core genera revealed context-dependent dynamics in the cattle gut microbiome. *Bacteroides* spp. maintained a stable presence across treatments and years, broadly consistent with the resilience of Bacteroidetes polysaccharide-degradation machinery described across gut environments (Jin et al., 2024; McKee et al., 2021). Notably, its abundance peaked in August 2023 in Std-Con pastures when *Faecalibacterium* spp. declined, suggesting a possible shift in fibre-degrading activity under winter conditions with lower availability of fermentable substrates. *Bacteroides* spp. are recognised for versatile carbohydrate metabolism and capacity to adapt to fluctuating nutrient environments and may increase in abundance when other fibre-degrading taxa are less active or absent, thus helping to maintain gut fibre metabolism under suboptimal dietary conditions (McKee et al., 2021). Other fibre- and starch-degrading taxa, within genus *Ruminococcus* spp. were enriched in cattle grazing on diverse regenerative pastures, consistent with varied substrate availability under mixed swards and with the synergistic roles of rumen and hindgut microorganisms described in ruminant lignocellulose degradation generally (Fu et al., 2025) and comparable substrate-driven enrichment of these genera has also been reported specifically in the rumen microbial community (Russell and Rychlik, 2001) (Figure 1).

In sheep, *Ruminococcus* spp. were prominent alongside *Faecalibacterium* spp. across treatments. Fibrolytic *Ruminococcus* spp. activity is well characterised in the rumen, where it supports breakdown of plant cell wall cellulose (Koike and Kobayashi, 2009), and a similar role is plausible in the hindgut community (Figure 2). Notably, sporadic peaks assigned to *C. perfringens*, particularly in Div-Reg in later spring (November 2023), may reflect the effect of seasonal shifts in rapidly growing, carbohydrate-rich pasture during late spring, which provides an ideal substrate for proliferation of opportunistic pathogenic taxa in the gastrointestinal tract generally (Grenda et al., 2023). This seasonal nutrient surge and dietary change can disrupt gut microbial equilibrium, creating opportunities for pathogenic bacteria to flourish, a dynamic also demonstrated experimentally using rumen-simulation systems challenged with *C. perfringens* (Wetzels et al., 2018). *Clostridium perfringens* specifically can cause severe ruminant diseases including haemorrhagic enteritis and abomasitis, particularly when transient blooms arise due to seasonal effects on pastures (Simpson et al., 2018). However, given the species-level resolution limits noted in Methods, this assignment is treated as indicative, and the pattern is interpreted as a probable *C. perfringens*-associated bloom. The intermittent detection of taxa such as *Parabacteroides* spp. and *Treponema* spp. suggests stochastic and substrate-driven responses to seasonal variation in fibre quality within the gut. This turnover among low-abundance taxa occurred alongside a stable core microbiome, consistent with findings that microbiome stability supports ruminant adaptation to seasonal change (Guo et al., 2024).

### 4.2 Microbial interactions and network patterns

Network analysis, restricted to a single treatment at one time point during winter, was used to explore co-occurrence patterns among dominant hindgut taxa during the dietary constraint period. As noted in Section 3.2, none of these correlations remained significant after FDR correction. Therefore, the patterns discussed below are interpreted as hypothesis-generating rather than as evidence of functional interaction.

In cattle, the strongest positive correlation coefficient was observed between two dominant fibre-fermenting taxa, *Faecalibacterium* spp. and *Bacteroides* spp. (Figures 4A,B). The co-occurrence observed here is consistent with, a cross-feeding relationship reported for these taxa in other systems (Culp and Goodman, 2023), but this remains a hypothesis rather than a demonstrated finding in the present study.

A comparable pattern was observed in sheep, where a moderate positive association was detected between *Prevotella* spp. and *Faecalibacterium* spp. *Prevotella* particularly *P. copri*, is known to degrade dietary polysaccharides and proteins into fermentation products such as succinate, acetate, and formate (Franke and Deppenmeier, 2018). These products may serve as substrates for *Faecalibacterium* spp. As above, this association reflects a compositional pattern rather than confirmed cross-feeding in this hindgut dataset.

In contrast, negative associations were consistently detected between *C. perfringens* and the core genera *Faecalibacterium* spp. and *Bacteroides* spp. in both ruminants (Figures 4A,B), consistent with its opportunistic pathogen status (Grenda et al., 2023; Songer and Miskimmins, 2004; Uzal and Songer, 2008). This negative association may reflect community instability associated with *C. perfringens* proliferation, though this cannot be confirmed from compositional data alone.

### 4.3 Effects of pasture management on microbial diversity

A prominent finding of this study is that cattle grazing on diverse regenerative pastures (Div-Reg) exhibited pronounced peaks in Shannon diversity in August 2023, the coldest winter month of the experimental period. Although regenerative practices may alter sward composition (Cosgrove et al., 2024), in this study this may have been offset by the greater botanical diversity of diverse pastures, which may have provided a wider range of fermentable substrate types, supporting multiple microbial niches rather than favouring a single dominant taxon (Li et al., 2023). This may explain the more even distribution of taxa and higher Shannon diversity observed, despite the typically restricted substrate availability during the winter period (Cui et al., 2023). By enhancing sward recovery and structural complexity, regenerative practices may further increase dietary heterogeneity, enriching the nutritional and biochemical diversity of forage available to livestock (Jezequel et al., 2024). In both management systems, spring pastures (October 2022) typically exhibited higher Shannon diversity, possibly reflecting the seasonal nutrient surge and the greater variety of plant species and nutritional components present during this period. Higher microbial diversity is expected to improve functional redundancy and metabolic flexibility, supporting more efficient use of spring forage and a more stable hindgut community during rapid dietary change (Belanche et al., 2023).

In sheep, pasture diversity did not correspond to detectable differences in gut microbiome diversity. Shannon diversity was elevated in both regenerative management treatments (Std-Reg and Div-Reg) in November 2023, and may reflect the enhanced pasture growth during spring, accelerated by regenerative practices of rotational grazing with longer recovery periods (Cosgrove et al., 2024; Jayaneththi et al., 2026a). This indicates that the diversity of the sheep gut microbiome may be less sensitive to pasture diversity, but it responds positively to seasonal fluctuations in forage availability and quality associated with regenerative management. This interpretation is consistent with an exploratory two-way ANOVA, which found no significant main effects of diversity or management, nor a significant interaction between them, on overall Shannon diversity in sheep as treatments in this study reflect integrated management systems rather than independently controlled factors, this result is best interpreted descriptively. The significant differences observed were specific to particular time points rather than reflecting a sustained treatment effect. The weaker treatment response in sheep may reflect differences in grazing behaviours. Sheep are typically more selective grazers than cattle, using their narrow muzzle to pick preferred plant species and parts even within diverse swards, whereas cattle graze more indiscriminately (Xu et al., 2024). This selective grazing may buffer actual dietary intake of sheep against pasture treatment differences, translating into weaker microbial differences than in cattle. Differences in rumen physiology between species may also play a role, though direct evidence for this is currently lacking.

Pairwise comparisons of beta diversity across years revealed significant effects of most comparative pasture management treatments (Std-Con vs. Div-Reg) on the hindgut microbiome. These effects were most pronounced between December 2024 and April 2025 in cattle, a period characterised by warm mean temperatures (17.6 °C) and a high mean soil moisture deficit (107 mm; NIWA, 2025), indicating that summer conditions that influence pasture growth and nutrient profiles can create more diverse feeding environments, which amplify differences in gut microbial community composition between pasture management systems (Guo et al., 2024; He et al., 2024; Jayaneththi et al., 2026a). Sheep showed similar effects, with the most pronounced contrasts occurring from late winter to early spring (August to October 2023), a period characterised by moderate rainfall (86–101 mm month^−1^) and rising mean temperatures (8.0 °C in August to 13.3 °C in October 2023; NIWA, 2025) and vigorous pasture growth. These results are broadly consistent with evidence that sward diversity, grazing management, and seasonal shifts in forage availability shape gut microbial community composition in sheep, both in the rumen (Fan et al., 2021) and hindgut (Jefferson et al., 2025; Li et al., 2025).

These diversity patterns are consistent with the hypothesis that diverse, regeneratively managed pastures may support more compositionally rich, and potentially more stable, gut microbial communities compared with standard contemporary systems in grazing livestock.

However, despite these insights, this study has several limitations, as with any research conducted within a working, multi-year farm system rather than a fully controlled experimental setting. The 16S rRNA gene amplicon sequencing does not quantify functional genes and metabolic pathways. Consequently, functional roles attributed to key taxa are inferred from existing literature and ecological patterns rather than measured directly. Furthermore, 16S sequencing of the V3-V4 region cannot reliably classify taxa to species level, derived species names are therefore retained only in consideration of the descriptive nature of this study and should be interpreted as indicative rather than definitive. Additionally, taxonomic assignments were performed using the Greengenes 16S database (release 13_5), which, while widely used, predates more recent reference databases such as SILVA or GTDB, limiting interpretations from these data. The microbial networks were constructed from a subset of samples and should be interpreted as exploratory. This study did not directly measure animal production or health outcomes (e.g., liveweight gain, milk yield, or body condition score) alongside the microbiome dataset, limiting the ability to directly link microbiome composition to host performance outcomes within this analysis. A companion analysis of milk production at the same dairy site found no significant difference in total lactation yield between these same three treatments (Moreno-González et al., 2025), suggesting that the compositional differences in gut microbiota observed here did not correspond to a detectable production outcome in this instance, though direct linkage was not tested. Similarly, empirical forage chemical composition data (e.g., NDF, ADF, crude protein, water-soluble carbohydrates, tannin content) were not collected, limiting the ability to directly test substrate-availability explanations for the observed microbiome shifts. Quantitative faecal short-chain fatty acid analysis was also not performed, and soil nutrient data were also not collected, making it difficult to determine whether seasonal microbiome shifts were driven by climate or by forage quality.

Future work combining long-read 16S sequencing or shotgun metagenomics with RNA-seq-based functional annotation, alongside animal production and health monitoring, forage-quality sampling, faecal short-chain fatty acid quantification, and soil nutrient data within a similar longitudinal design, would help confirm the functional consequences of the observed shifts in community composition and co-occurrence patterns.

## 5 Conclusion

This multi-year study demonstrates that, although the ruminant gut microbiome remains broadly stable over time, its finer-scale temporal dynamics are strongly shaped by pasture management systems, with seasonal changes in pasture conditions playing a central role in structuring gut microbial communities. Diverse pastures and regenerative management were associated with higher microbial diversities at specific seasonal time points and tended to favour dominant and co-dominant taxa. These patterns suggest that diverse, regeneratively managed pastures may support a more compositionally rich gut microbial community. As this study was conducted under real, farm-scale management conditions over multiple years, it provides a valuable foundation for understanding these dynamics under practical grazing systems. However, the specific effects of these compositional shifts on ruminant health or productivity remain to be established through further research.

## Data Availability

The datasets generated and analysed during the current study are available in the European Nucleotide Archive repository (https://www.ebi.ac.uk/ena/browser/view/PRJEB105277) (Jayaneththi et al., 2026c). This dataset includes the sequencing reads generated from 16S rRNA gene sequencing. No custom scripts were generated for this study.

## Footnotes

1
https://fbreitwieser.shinyapps.io/pavian/
