# Supplementary material for: Pasture diversity and regenerative management shape ruminant gut microbiome dynamics within a real multi-year farming system
Source: Front Microbiol. 2026 Aug 31;17:1928530. doi: 10.3389/fmicb.2026.1928530 (PMC13572636; doi:10.3389/fmicb.2026.1928530)
Supplement: Supplementary file 1 [file Table_1.DOCX]

**Supplementary Material**

Table S1. Estimated marginal means (EMMeans, %) ± SE of Faecalibacterium prausnitzii and *Bacteroides* *uniformis* relative abundances in cattle across treatments and sampling time points.

| **Time Point** | ***Faecalibacterium prausnitzii*** | | | ***Bacteroides uniformis*** | | |
| --- | --- | --- | --- | --- | --- | --- |
|  | **Std-Con** | **Div-Reg** | **Div-Con** | **Std-Con** | **Div-Reg** | **Div-Con** |
| Aug 2022 | 32.1±2.8^ab^ | 41.0±2.86^a^ | 38.8±2.86^ab^ | 20.4±1.35^bc^ | 21.2±1.35^ab^ | 19.7±1.35^ab^ |
| Oct 2022 | 27.1±2.86^ab^ | 28.6±2.86^abcde^ | 30.6±2.86^abc^ | 25.7±1.35^b^ | 25.2±1.35^a^ | 23.2±1.35^ab^ |
| Nov2022 | 29.1±2.86^ab^ | 35.9±2.86^abc^ | 32.5±2.86^abc^ | 24.7±1.35^b^ | 22.9±1.35^a^ | 23.7±1.35^ab^ |
| Dec 2022 | 27.2±2.86^ab^ | 23.9±2.86^cde^ | 27.4±2.86^abc^ | 20.1±1.35^bc^ | 19.9±1.35^ab^ | 21.6±1.35^ab^ |
| Feb 2023 | 19.8±2.86^b^ | 21.6±2.86^de^ | 23.8±2.86^c^ | 19.9±1.35^bc^ | 23.4±1.35^a^ | 21.0±1.35^ab^ |
| Apr 2023 | 26.7±3.68^ab^ | 19.8±3.68^cde^ | 28.6±3.68^abc^ | 13.5±1.74^c^ | 14.2±1.74^b^ | 18.2±1.74^b^ |
| Aug 2023 | - | 17.8±3.68e | 43.0±3.68^a^ | 35.5±1.74^a^ | 24.5±1.74^a^ | 23.6±1.74^ab^ |
| Oct 2023 | 35.2±2.86^a^ | 28.7±2.86^abcde^ | 28.2±2.86^abc^ | 23.9±1.35^b^ | 24.7±1.35^a^ | 25.6±1.35^ab^ |
| Nov 2023 | 20.0 ± 2.86^b^ | 25.1±2.86^bcde^ | 26.0±2.86^bc^ | 24.5±1.35^b^ | 25.7±1.35^a^ | 26.1±1.35^a^ |
| Dec 2023 | 21.7± 3.02^ab^ | 21.9±3.02^cde^ | 23.2±3.02^c^ | 21.3±1.42^bc^ | 21.4±1.42^ab^ | 21.0±1.42^ab^ |
| Feb 2024 | 35.1±3.02^a^ | 19.4±3.02^e^ | 27.8±3.02^abc^ | 23.8±1.42^b^ | 21.7±1.42^ab^ | 23.0±1.42^ab^ |
| Apr 2024 | 23.2±2.86^ab^ | 38.7±2.86^ab^ | 37.9±2.86^ab^ | 21.9±1.35^b^ | 21.7±1.35^ab^ | 22.4±1.35^ab^ |
| Aug 2024 | 22.0±3.02^ab^ | 36.2±3.02^abc^ | 35.8±3.02^abc^ | 25.9±1.42^b^ | 22.7±1.42^a^ | 23.4±1.42^ab^ |
| Oct 2024 | 30.0±3.02^ab^ | 34.6±3.02^abcd^ | 34.6±3.02^abc^ | 21.8±1.42^b^ | 21.7±1.42^ab^ | 22.0±1.42^ab^ |
| Nov 2024 | 27.7±2.86^ab^ | 29.4±2.86^abcde^ | 27.6±2.86^abc^ | 22.2±1.35^b^ | 22.4±1.35^a^ | 22.4±1.35^ab^ |
| Dec 2024 | 23.9±3.02^ab^ | 26.5±3.02^bcde^ | 28.3±3.02^abc^ | 21.8±1.42^b^ | 22.3±1.42^a^ | 22.3±1.42^ab^ |
| Feb 2025 | 24.9±2.86^ab^ | 29.4±2.86a^bcde^ | 34.2±2.86^abc^ | 21.4±1.35^b^ | 22.5±1.35^a^ | 21.3±1.35^ab^ |
| Apr 2025 | 36.2±3.02^a^ | 36.2±3.02^abc^ | 32.2±3.02^abc^ | 20.3±1.42^bc^ | 20.8±1.42^ab^ | 22.2±1.42^ab^ |

Missing values (–) indicate species below the reporting threshold, defined as either less than 50% of the highest observed mean relative abundance or below the Bracken read count threshold. Different letters indicate significant differences among timepoints within the same treatment (*p < 0.05*). Std-Con: standard pastures under contemporary management; Div-Reg: diverse pastures under regenerative management; Div-Con: diverse pastures under contemporary management.

Table S2: Estimated marginal means (EMMeans %) ± SE of *Faecalibacterium* relative abundances in sheep across treatments and sampling time points.

| **Time Point** | **Std-Con** | **Std-Reg** | **Div-Con** | **Div-Reg** |
| --- | --- | --- | --- | --- |
| Mar 2023 | 24.9±2.66^a^ | 21.3±2.66^a^ | 27.1±2.05^a^ | 27.3±2.06^ab^ |
| Jun 2023 | 19.9±2.06^ab^ | 29.4±2.06^a^ | 24.3±2.06^a^ | 24.6±2.06^ab^ |
| Aug 2023 | 25.3±2.06^a^ | 27.3±2.06^a^ | 26.1±2.06^a^ | 29.5±2.06^a^ |
| Oct 2023 | 28.4±2.06^a^ | 22.9±2.06^a^ | 26.0±2.06^a^ | 24.7±2.06^ab^ |
| Nov 2023 | 26.0±2.30^a^ | 20.0±2.30^a^ | 24.8±2.30^a^ | 18.1±2.31^b^ |
| Mar 2024 | 22.5±2.06^ab^ | 24.5±2.06^a^ | 22.9±2.06^a^ | 29.5±2.06^a^ |
| June 2024 | 26.8±2.07^a^ | 25.5±2.06^a^ | 28.9±2.06^a^ | 27.1±2.06^ab^ |
| Aug 2024 | 28.3±2.06^a^ | 27.4±2.06^a^ | 26.9±2.06^a^ | 25.0±2.06^ab^ |
| Oct 2024 | 22.4±2.31^ab^ | 29.8±2.06^a^ | 27.2±2.30^a^ | 25.4±2.31^ab^ |
| Nov 2024 | 13.2±2.66^b^ | 22.1±2.66^a^ | 27.2±2.66^a^ | 29.4±2.66^a^ |

Different letters indicate significant differences among timepoints within the same treatment (*p < 0.05*). Std-Con: standard pastures under contemporary management; Std-Reg: standard pastures under regenerative management; Div-Con: diverse pastures under contemporary management; Div-Reg: diverse pastures under regenerative management.

Table S3. Estimated marginal means (EMMeans) ± SE of Shannon index in cattle across treatments and sampling time points.

| **Time Point** | **Std-Con** | **Div-Reg** | **Div-Con** |
| --- | --- | --- | --- |
| Aug 2022 | 2.19±0.065^c^ | 1.82±0.065^c^ | 2.01±0.084^cde^ |
| Oct 2022 | 2.43±0.065^a^ | 2.43±0.065^a^ | 2.42±0.065^a^ |
| Nov2022 | 2.42±0.065^a^ | 2.20±0.065^ab^ | 2.31±0.065^abcd^ |
| Dec 2022 | 2.04±0.065^bc^ | 2.09±0.065^bc^ | 2.03±0.065^cd^ |
| Feb 2023 | 2.31±0.065^abc^ | 2.20±0.065^ab^ | 2.26±0.065^abcd^ |
| Apr 2023 | 2.40±0.084^ab^ | 2.35±0.084^ab^ | 2.38±0.084^abc^ |
| Aug 2023 | 1.63±0.084^d^ | 2.33±0.084^ab^ | 1.65±0.084^e^ |
| Oct 2023 | 2.03±0.065^bc^ | 2.13±0.065^abc^ | 2.06±0.065^bcd^ |
| Nov 2023 | 2.11±0.065^abc^ | 2.22±0.065^ab^ | 2.33±0.065^abc^ |
| Dec 2023 | 2.16±0.069^abc^ | 2.13±0.069^abc^ | 2.29±0.069^abcd^ |
| Feb 2024 | 2.12±0.069^abc^ | 2.17±0.069^ab^ | 2.12±0.069^abcd^ |
| Apr 2024 | 2.28±0.065^abc^ | 2.05±0.065^bc^ | 1.99±0.065^de^ |
| Aug 2024 | 2.20±0.069^abc^ | 2.10±0.069^abc^ | 2.20±0.069^abcd^ |
| Oct 2024 | 2.33±0.069^abc^ | 2.27±0.069^ab^ | 2.29±0.069^abcd^ |
| Nov 2024 | 2.29±0.065^abc^ | 2.33±0.065^ab^ | 2.36±0.065^ab^ |
| Dec 2024 | 2.36±0.069^ab^ | 2.14±0.069^abc^ | 2.17±0.069^abcd^ |
| Feb 2025 | 2.13±0.065^abc^ | 2.22±0.065^ab^ | 2.20±0.065^abcd^ |
| Apr 2025 | 2.14±0.069^abc^ | 2.13±0.069^abc^ | 2.18±0.069^abcd^ |

Different letters indicate significant differences among time points within the same treatment (*p* < 0.05). Std-Con: standard pastures under contemporary management; Div-Reg: diverse pastures under regenerative management; Div-Con: diverse pastures under contemporary management.

Table S4. Estimated marginal means (EMMeans) ± SE of Shannon index in sheep across treatments and sampling time points.

| **Time Point** | **Std-Con** | **Std-Reg** | **Div-Con** | **Div-Reg** |
| --- | --- | --- | --- | --- |
| Mar 2023 | 2.38±0.068^a^ | 2.24±0.068^b^ | 2.34±0.068^a^ | 2.19±0.068^b^ |
| Jun 2023 | 2.33±0.068^a^ | 2.27±0.068^ab^ | 2.44±0.068^a^ | 2.35±0.068^ab^ |
| Aug 2023 | 2.31±0.068^a^ | 2.36±0.068^ab^ | 2.31±0.068^a^ | 2.19±0.068^ab^ |
| Oct 2023 | 2.42±0.068^a^ | 2.45±0.068^ab^ | 2.41±0.068^a^ | 2.37±0.068^ab^ |
| Nov 2023 | 2.54±0.077^a^ | 2.59±0.076^a^ | 2.43±0.076^a^ | 2.52±0.077^a^ |
| Mar 2024 | 2.44±0.068^a^ | 2.49±0.068^ab^ | 2.45±0.068^a^ | 2.34±0.068^ab^ |
| June 2024 | 2.27±0.068^a^ | 2.36±0.068^ab^ | 2.36±0.068^a^ | 2.38±0.068^ab^ |
| Aug 2024 | 2.57±0.068^a^ | 2.36±0.068^ab^ | 2.50±0.068^a^ | 2.37±0.068^ab^ |
| Oct 2024 | 2.41±0.077^a^ | 2.37±0.077^ab^ | 2.47±0.076^a^ | 2.42±0.077^ab^ |

Different letters indicate significant differences among time points within the same treatment (*p* < 0.05). Std-Con: standard pastures under contemporary management; Std-Reg: standard pastures under regenerative management; Div-Con: diverse pastures under contemporary management; Div-Reg: diverse pastures under regenerative management.
